# Tuberculosis and risk of cancer: A systematic review and meta-analysis

**DOI:** 10.1371/journal.pone.0278661

**Published:** 2022-12-30

**Authors:** Pauline Luczynski, Philip Poulin, Kamila Romanowski, James C. Johnston

**Affiliations:** 1 Department of Medicine, University of British Columbia, Vancouver, British Columbia, Canada; 2 Provincial TB Services, British Columbia Centre for Disease Control, Vancouver, Canada; Institut d’Investigacio Biomedica de Bellvitge, SPAIN

## Abstract

**Introduction:**

Cancer is a major cause of death among people who experience tuberculosis (TB), but little is known about its timing and incidence following TB treatment. Our primary objectives were to estimate the pooled risk of all and site-specific malignancies in people with TB compared to the general population or suitable controls. Our secondary objective was to describe the pooled risk of cancer at different time points following TB diagnosis.

**Methods:**

This study was prospectively registered (PROSPERO: CRD42021277819). We systematically searched MEDLINE, Embase, and the Cochrane Database for studies published between 1980 and 2021. We included original observational research articles that estimated cancer risk among people with TB compared to controls. Studies were excluded if they had a study population of fewer than 50 individuals; used cross-sectional, case series, or case report designs; and had a follow-up period of less than 12 months. Random-effects meta-analysis was used to obtain the pooled risk of cancer in the TB population.

**Results:**

Of the 5,160 unique studies identified, data from 17 studies were included. When compared to controls, the pooled standardized incidence ratios (SIR) of all cancer (SIR 1.62, 95% CI 1.35–1.93, I^2^ = 97%) and lung cancer (SIR 3.20, 95% CI 2.21–4.63, I^2^ = 90%) was increased in the TB population. The pooled risk of all cancers and lung cancer was highest within the first year following TB diagnosis (SIR 4.70, 95% CI 1.80–12.27, I^2^ = 99%) but remained over five years of follow-up.

**Conclusions:**

People with TB have an increased risk of both pulmonary and non-pulmonary cancers. Further research on cancer following TB diagnosis is needed to develop effective screening and early detection strategies. Clinicians should have a high index of suspicion for cancer in people with TB, particularly in the first year following TB diagnosis.

## Introduction

Over 10 million people are affected by tuberculosis disease (TB) each year, with an estimated 155 million TB survivors globally in 2020 [1, 2]. TB survivors face increased all-cause mortality [3] and reduced life expectancy [4], which is thought to be due in part to the higher risk of non-communicable diseases following TB [5, 6]. In response, there is a growing movement to integrate non-communicable disease prevention and management in the care of TB survivors [7]. This includes cancer, which is second only to cardiovascular disease as the leading cause of death in this population [3].

Infectious pathogens are known carcinogens, causing an estimated 2.2 million cancers globally each year [8]. It has long been hypothesized that infection and disease from *Mycobacterium tuberculosis*, the bacteria causing TB, could increase the risk of cancer [9]. Moreover, people with TB are disproportionately affected by risk factors for malignancy such as smoking, immune suppression, and alcohol misuse [10]. Recent systematic reviews have shown an association between TB and both pulmonary [11] and non-pulmonary cancers [12]. Despite this link, there are currently no guidelines for cancer screening in TB populations. A clear understanding of the incidence and timing of cancer following TB is necessary to guide clinical practice and to potentially develop effective cancer screening and prevention strategies. We therefore systematically reviewed the published literature on TB and cancer as a logical step toward evidence-based guidance.

For this systematic review and meta-analysis, our primary objectives were to estimate the pooled risk of overall malignancy and site-specific malignancies in people with TB compared to the general population or suitable controls. Our secondary objective was to describe the pooled risk of cancer diagnosis at different time points following TB diagnosis.

## Materials and methods

This study was performed following the Preferred Reporting Items for Systematic Review and Meta-Analyses (PRISMA) guidelines [13] and prospectively registered on PROSPERO (registration number CRD42021277819).

### Identification of studies

We systematically searched MEDLINE, Embase, and the Cochrane Database of Systematic Reviews for articles published from January 1^st^, 1980 to September 15^th^, 2021. We used a broad search strategy with medical subject headings (MeSH) and text words. The full search strategy is presented in S1A–S1C Table. Potentially relevant articles were selected for a full-text review. A manual search of reference lists of eligible full-text articles was also performed.

### Inclusion and exclusion criteria

Studies were included if they met all of the following criteria: original research papers with prospective or retrospective observational, or case-control study designs; included bacteriological or clinical confirmation of TB; included histologic or clinical confirmation of cancer; reported a risk estimate of one or more cancer type among people with TB compared to the general population or suitable controls; and used study-defined controls without TB accounting for at least one demographic (e.g. age or sex) or medical risk factor (e.g. smoking status). A clinical diagnosis of TB includes diagnosis by a physician, ICD code, or by health administrative database. Studies were excluded if they had a study population of less than 50 individuals; used a cross-sectional, case series, or case report design; had a follow-up period of less than 12 months; or were not available in English.

Two authors (PP and PL) screened titles and abstracts of studies identified by the search. Relevant studies then underwent full-text review, applying our inclusion and exclusion criteria. Any uncertainties or disagreements regarding the inclusion or exclusion of articles was settled by a third reviewer (JJ). When multiple articles from the same data sources reported duplicated data, the study with the most complete reporting of outcomes was included.

### Data extraction

The items for data extraction were predefined and agreed upon by all authors. Variables were extracted and coded by two independent reviewers (PP and PL) using a common template. From each study, we extracted the first author’s surname, year of publication, duration of following, study location, and source of diagnosis. We also extracted population characteristics, including study size, and details of TB and cancer diagnosis, outcome measures including 95% confidence intervals, and the adjustment variables.

### Quality assessment

Two reviewers (PP and PL) independently assessed the included studies for risk of bias (ROB) using the Risk of Bias in Non-randomized Studies of Interventions (ROBINS-I) tool [14]. Using this tool, we incorporated anticipated confounders into a predefined criteria for each domain of bias (S2 Table). Major methodological concerns resulting in a serious ROB included lack of control for smoking, TB diagnosed by radiograph only, a high rate of missing data, and unadjusted outcome measures. We assessed the ROB uniformly across studies but excluded ROB from deviation from intended intervention. The lowest score in any subgroup was used to give an overall bias rating.

### Outcomes

The primary endpoint of this study was pooled risk of pulmonary and non-pulmonary cancers in people with TB compared to control populations. The secondary endpoints were examining the effect of age, sex, and smoking on cancer risk as well as determining the pooled risk of cancer diagnosis at different time points following TB diagnosis.

### Statistical analysis

From eligible studies, we extracted incidence and standardized incidence ratios (SIR), or we calculated them when data was sufficient. For studies where we calculated SIRs, we determined the 95% CIs according to the formula described by Rothman and Greenland [15]. For studies that did not report SIRs, we extracted either adjusted hazard ratios (aHR), adjusted odds ratios (aOR) or rate ratios (RR); aORs and RRs were pooled with SIRs, and aHRs were pooled separately, in line with recommended practice [16]. Details on the aHRs used in this meta-analysis are found in S3 Table.

Once extracted, estimates were log-transformed, and the 95% CIs were used to calculate corresponding standard errors. Data were then pooled using generic inverse variance random-effects models. A random-effects model was chosen given the significant diversity and heterogeneity seen in observation studies [16, 17]. The pooled log-effect estimates were then back-transformed for interpretation.

For our primary and secondary analyses, we included all studies without overlapping populations. In studies with overlapping populations, we included the study with the lowest risk of bias. If the risk of bias was equal, we included the study with the largest sample population. Site-specific malignancies were combined only when data from ≥ 3 studies were included for a given cancer type. For our secondary analysis, we pooled effect measures by time periods of less than one year, between one year and five years, and greater than five years.

We estimated the proportion of total variability due to between-study heterogeneity using I^2^ [18, 19] and classified it as low-level (<25%), moderate-level (25–49%), substantial level (50–74%), or high-level (>75%). In the event of high heterogeneity and sufficient data, we conducted subset analyses removing one or more study to investigate the source.

We conducted sensitivity analyses removing ORs from the pooled estimate, where there was sufficient data. All analyses were conducted in R (V.4.1.2) [20].

## Results

### Literature search and study characteristics

**Fig 1:**
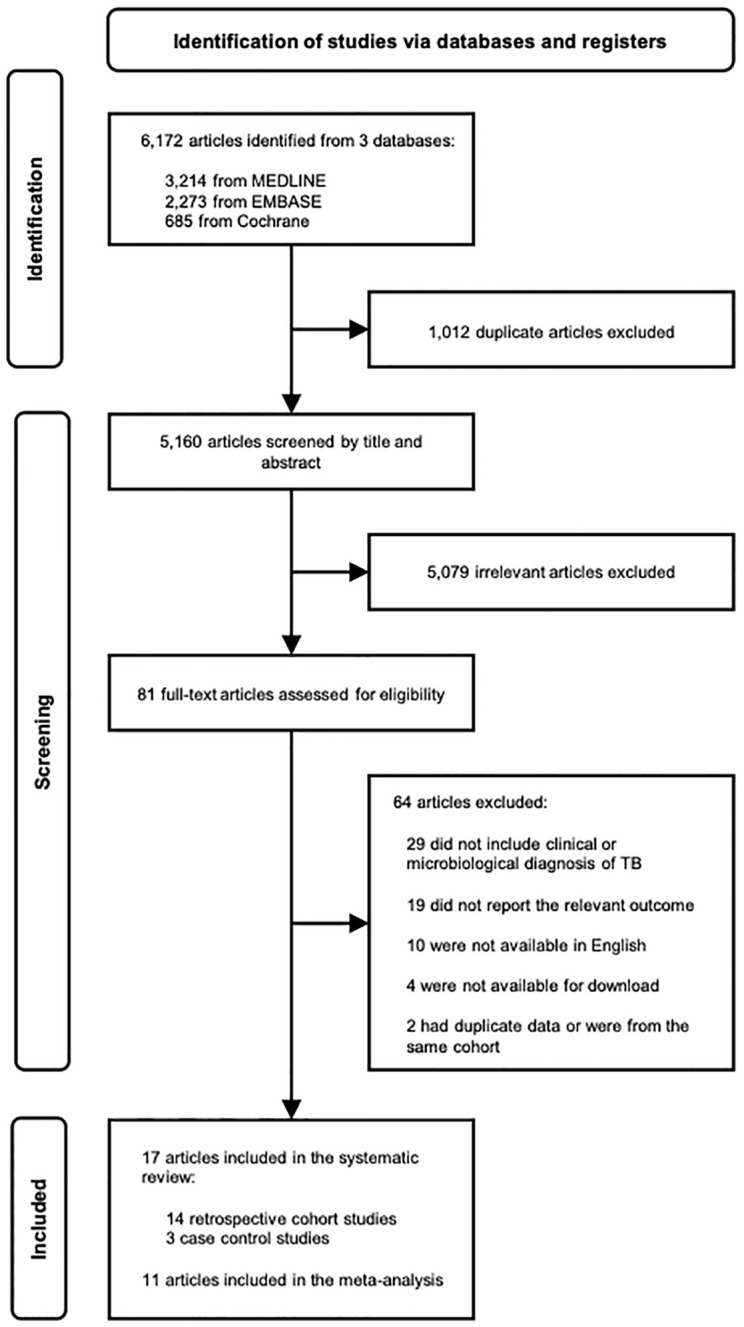
PRISMA flow diagram. PRISMA flow diagram for articles on tuberculosis and risk of cancer. PRISMA = Preferred Reporting Items for Systematic Reviews and Meta-Analyses.

We identified 5,160 unique articles in our search, of which 81 qualified for full-text review (Fig 1). Seventeen studies were included in our systematic review: fourteen retrospective cohort studies [21–34] and three case-control studies [35–37]. Study characteristics are summarized in Table 1. The included studies were published from 1992 to 2021. All studies were from high or upper-middle income countries. Fourteen studies described individuals with active TB and cancer from national registries or databases, two from hospitals, and one from a private insurance database. Further details of TB diagnosis and treatment for included studies are summarized in S5 Table and further details of cancer diagnosis for included studies are summarized in S6 Table.

**Table 1:** Characteristics of included studies for systematic review of tuberculosis and risk of cancer.

Author	Study design	Country, WHO region	Total number of participants	Number of patients with TB	Study group	Comparison group	Source of TB diagnosis	Source of cancer diagnosis	Duration of follow-up	Outcome measure
Doody *et al*,. 1992 [35]	Case-control	United States, AMR	1,361	83	Adults with hematologic malignancies	Controls matched by region, sex, age, and insurance membership period	Kaiser Permanente records for California and Oregon, USA (1956–1982)	Kaiser Permanente records for California and Oregon, USA (1956–1982)	Up to 27 years	aOR
Askling *et al*., 2001 [26]	Retrospective cohort	Sweden, EUR	5,050	5,050	Patients diagnosed and/or admitted to hospital with TB	Expected controls calculated using sex-, age-, and calendar period-specific incidence rates	Two tuberculosis dispensaries and one sanitorium in Sweden (1939–1960)	Swedish Cancer Register (1958–1996)	Up to 21 years	SIR
Yu *et al*., 2011 [21]	Retrospective cohort	Taiwan, WPR	716,872	4,480	Adults with newly diagnosed pulmonary TB	Adults without TB	NHI program research database of Taiwan (1998–2001)	NHI program research database of Taiwan (1998–2007).	7 to 9 years	aHR
Wu *et al*., 2011 [22]	Retrospective cohort	Taiwan, WPR	29,641	5,657	Patients with active pulmonary TB	Controls without TB matched by age and sex within the same period	NHI program research database of Taiwan (1997–2008)	Registry for Catastrophic Illness Patient Database of Taiwan (1997–2008)	1 to 12 years with a mean of 6 years	aHR
Shiels *et al*., 2011 [27]	Retrospective cohort	Finland, EUR	29,133	273	Adult male smokers with hospital-treated pulmonary TB	Adult male smokers without TB	National Hospital Discharge Register of Finland (1976–1995)	Finnish Cancer Registry (1985–2005)	Up to 20 years	aHR
Kuo *et al*., 2013 [28]	Retrospective cohort	Taiwan, WPR	6,699	6,699	Adults with newly diagnosed TB	Expected controls calculated using sex-, age-, and calendar period-specific incidence rates	NHI program research database of Taiwan (2000–2010)	Registry for Catastrophic Illness Patient Database of Taiwan (2000–2010)	Up to 10 years with a median of 3.8 years.	SIR
Lien *et al*., 2013 [29]	Retrospective cohort	Taiwan, WPR	Not reported	135,142	Patients with newly diagnosed active TB	Patients without TB	NHI program research database of Taiwan (1998–2008)	Registry for Catastrophic Illness Patient Database of Taiwan (1998–2010)	Up to 12 years with a median of 7.1 years and mean of 7.0 years	IR
Simonsen *et al*., 2014 [30]	Retrospective cohort	Denmark, EUR	15,024	15,024	Patients with newly diagnosed active TB	Expected controls calculated using sex-, age-, and year of diagnosis-specific incidence rates	Danish National Registry of Patients (1978–2011)	Danish Cancer Registry (1978–2011)	Up to 34 years with a median of 8.5 years	SIR
Kristinsson *et al*., 2015 [36]	Case-control	Sweden, EUR	36,654	68	Patients with Hodgkin lymphoma	Controls matched by sex, year of birth, and country of residence	Swedish Patient Registry (1964–2004)	Swedish Cancer Register (1965–2004)	Up to 40 years	aOR
Huang *et al*., 2015 [31]	Retrospective cohort	Taiwan, WPR	15,219,024	111,521	Adults with lung cancer	Adults without lung cancer	NHI program research database of Taiwan (2001–2003)	Taiwan Cancer Registry Database (2004–2008)	Up to 7 years	aHR
Everatt *et al*., 2016 [32]	Retrospective cohort	Lithuania, EUR	21,986	21,986	Adults with TB	Expected controls calculated using sex-, age-, and calendar period-specific incidence rates	Lithuanian TB registry (1998–2012)	Lithuanian Cancer Registry (1998–2012)	Up to 14 years	SIR
Hong *et al*., 2016 [34]	Retrospective cohort	Republic of Korea, WPR	1,607,710	79,298	Adults with pulmonary TB	Adults without TB	NHIS of Korea (1997–2013)	NHIS of Korea (1997–2013)	Up to 16 years	aHR
Everatt *et al*., 2017 [33]	Retrospective cohort	Lithuania, EUR	21,986	21,986	Adults with TB	Expected controls calculated using sex-, age-, and calendar period-specific incidence rates	Lithuanian TB registry (1998–2012)	Lithuanian Cancer Registry (1998–2012)	Up to 14 years	SIR
Oh *et al*., 2020 [23]	Retrospective cohort	Republic of Korea, WPR	20,252	2,640	Adults with pulmonary TB	Adults without TB	KNHANES database (2008–2013)	Korea Central Cancer Registry (2008–2013)	Up to 6 years with a mean of 4 years	aHR
An *et al*., 2020 [24]	Retrospective cohort	Republic of Korea, WPR	22,656	3,776	Adults with newly diagnosed pulmonary TB	Adult controls without TB matched by age and sex	Korean National Health Insurance Service-National Sample Cohort (2003–2013)	Korean National Health Insurance Service-National Sample Cohort (2003–2013)	Up to 11 years	aHR
Park *et al*., 2021 [25]	Retrospective cohort	Republic of Korea, WPR	47,632	5,325	Adults with pulmonary TB and COPD	Adult controls without COPD matched for age, sex, smoking history, and pulmonary TB status	Korean National Health Insurance-Service-National Sample Cohort 2.0 (2002–2015)	Korean National Health Insurance-Service-National Sample Cohort 2.0 (2002–2015)	Up to 13 years with a median of 7.7 years	aHR
Chen *et al*., 2021 [37]	Case-control	China, WPR	45,455	1,951	Patients with a malignant tumor	Patients diagnosed with a benign tumor	Department of TB Registry in Xinjiang Cancer Hospital in China (period not reported)	Xinjiang Cancer Hospital (2016–2018)	Not reported	aOR

aHR = adjusted hazard ratio, AMR = Region of the Americas, aOR = adjusted odds ratio, COPD = chronic obstructive pulmonary disease, EUR = European Region, NHI = National Health Insurance, NHIS = National Health Insurance Service, SIR = standardized incidence ratio, TB = tuberculosis, WPR = Western Pacific Region.

### Risk of bias

The ROB for included studies is presented in Table 2. The overall ROB was determined to be moderate in five studies [24, 27, 32–34] and serious in twelve studies [21–23, 26, 28–31, 35–38]. No studies had a critical risk of bias and, therefore, none were excluded from analyses [16].

**Table 2:** Assessment of risk of bias using the Risk of Bias in Non-randomized Studies of Interventions (ROBINS-I) tool.

Author	Confounding	Selection and intervention classification	Missing data	Measurement of outcome	Selection of reported result	Overall
Doody *et al*,. 1992 [35]	Serious	Moderate	Serious	Moderate	Moderate	Serious
Askling *et al*., 2001 [26]	Serious	Moderate	Low	Moderate	Moderate	Serious
Yu *et al*., 2011 [21]	Serious	Moderate	Moderate	Serious	Low	Serious
Wu *et al*., 2011 [22]	Serious	Moderate	Moderate	Moderate	Low	Serious
Shiels *et al*., 2011 [27]	Moderate	Moderate	Moderate	Moderate	Low	Moderate
Kuo *et al*., 2013 [28]	Serious	Moderate	Moderate	Moderate	Moderate	Serious
Lien *et al*., 2013 [29]	Serious	Moderate	Low	Moderate	Low	Serious
Simonsen *et al*., 2014 [30]	Serious	Low	Low	Moderate	Moderate	Serious
Kristinsson *et al*., 2015 [36]	Serious	Moderate	Moderate	Moderate	Moderate	Serious
Huang *et al*., 2015 [31]	Serious	Moderate	Moderate	Moderate	Low	Serious
Everatt *et al*., 2016 [32]	Moderate	Moderate	Moderate	Low	Low	Moderate
Hong *et al*., 2016 [34]	Moderate	Moderate	Moderate	Moderate	Low	Moderate
Everatt *et al*., 2017 [33]	Moderate	Moderate	Moderate	Moderate	Moderate	Moderate
Oh *et al*., 2020 [23]	Moderate	Serious	Moderate	Moderate	Low	Serious
An *et al*., 2020 [24]	Moderate	Moderate	Moderate	Moderate	Low	Moderate
Park *et al*., 2021 [25]	Moderate	Serious	Moderate	Moderate	Moderate	Serious
Chen *et al*., 2021 [37]	Serious	Moderate	Serious	Moderate	Moderate	Serious

### Overall risk of malignancy

**Fig 2:**
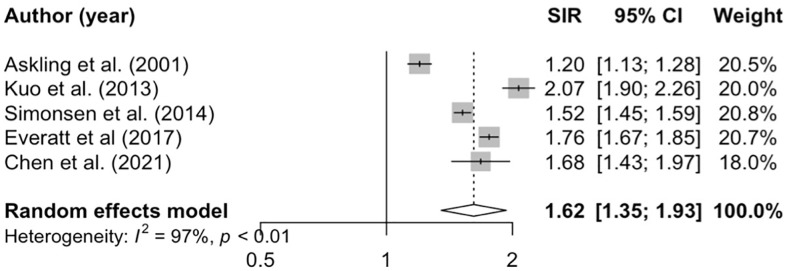
Malignancy risk. Meta-analysis of the SIR for all cancer among people with TB compared to age- and sex- standardized general populations of matched controls.

Five studies were included in our meta-analysis to estimate the overall pooled risk of malignancy following TB (Fig 2) [26, 28, 30, 33, 37]. The pooled SIR was 1.62 (95% CI 1.35–1.93, I^2^ = 97%; Fig 2). When data was stratified by sex, the pooled SIR of overall malignancy remained elevated in both males (SIR 1.64, 95% CI 1.16–2.33, I^2^ = 93%; S1A Fig) and females (SIR 1.58, 95% CI 1.31–1.91, I^2^ = 82%; S1B Fig).

Subset analyses removing studies to investigate sources of heterogeneity resulted in similar findings with high heterogeneity (results not shown). A sensitivity analysis including excluding ORs resulted in similar results (SIR 1.60, 95% CI 1.28–2.01, I^2^ = 98%), but heterogeneity remained high.

Subset analyses removing studies to investigate sources of heterogeneity resulted in similar findings with high heterogeneity (results not shown). A sensitivity analysis including excluding ORs resulted in similar results (SIR 1.60, 95% CI 1.28–2.01, I^2^ = 98%), but heterogeneity remained high.

### Risk of lung cancer

**Fig 3:**
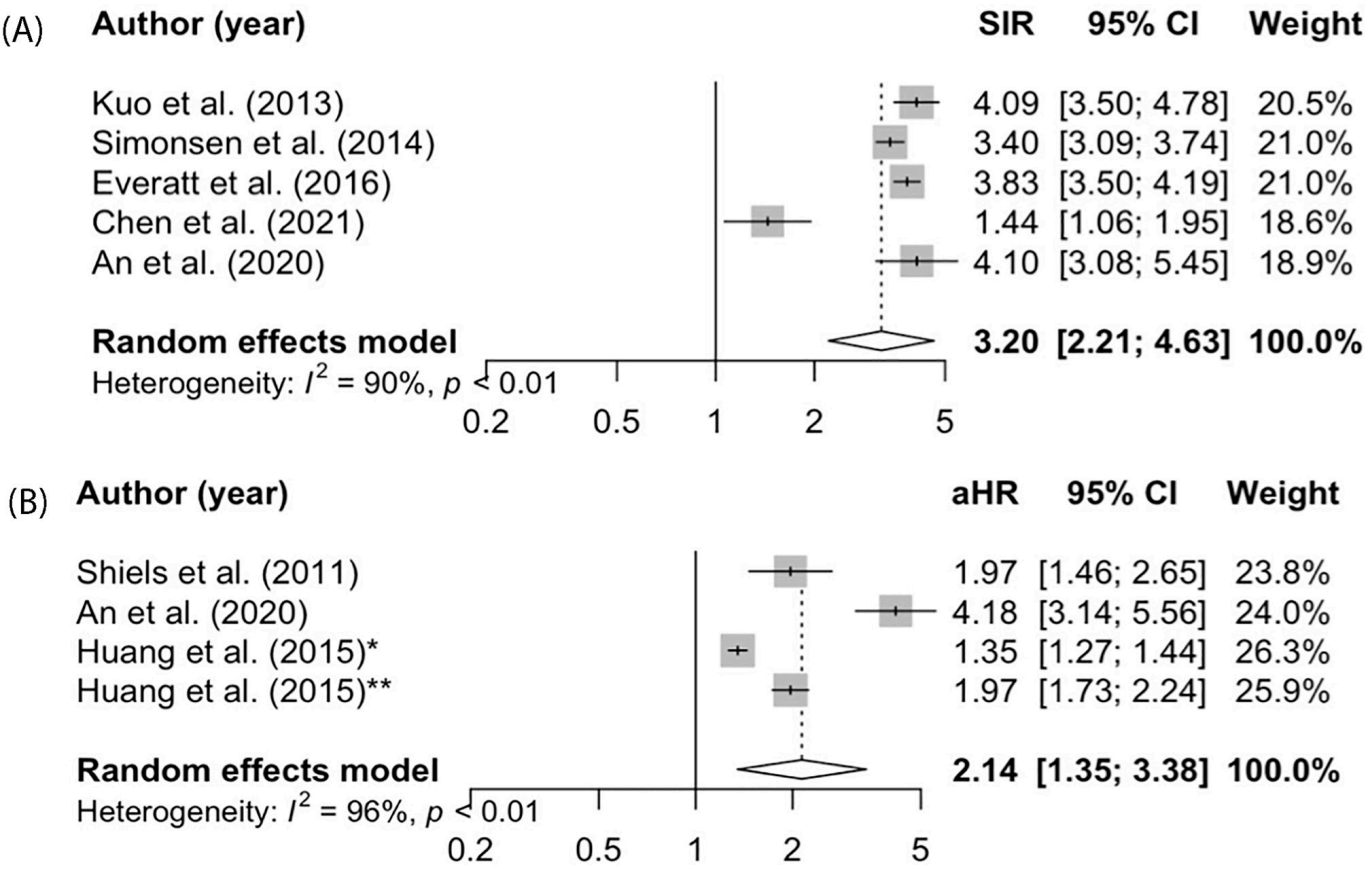
**A. Lung cancer (RR).** Meta-analysis of the SIR for lung cancer among people with TB compared to age- and sex- standardized general populations of matched controls. **B. Lung cancer (HR).** Meta-analysis of the adjusted hazard ratio for lung cancer among people with TB compared to matched controls.

Five studies were included in our meta-analysis to estimate the pooled SIR of lung cancer following TB [28, 30, 32, 37] and three studies were included to estimate the pooled aHR [24, 27, 31]. The pooled SIR was 3.20 (95% CI 2.21–4.63, I^2^ = 90%; Fig 3A) and the pooled aHR was 2.14 (95% CI 1.35–3.38, I^2^ = 96%; Fig 3B). When data was stratified by smoking, the pooled SIR for lung cancer remained elevated (SIR 3.09, 95% CI 2.22–4.29, I^2^ = 87%; S2 Fig). Sensitivity analysis excluding ORs resulted in a similar result, with lower heterogeneity (SIR 3.75; 95% CI 3.42–4.12, I^2^ = 47%).

### Risk of other cancer subtypes

**Fig 4:**
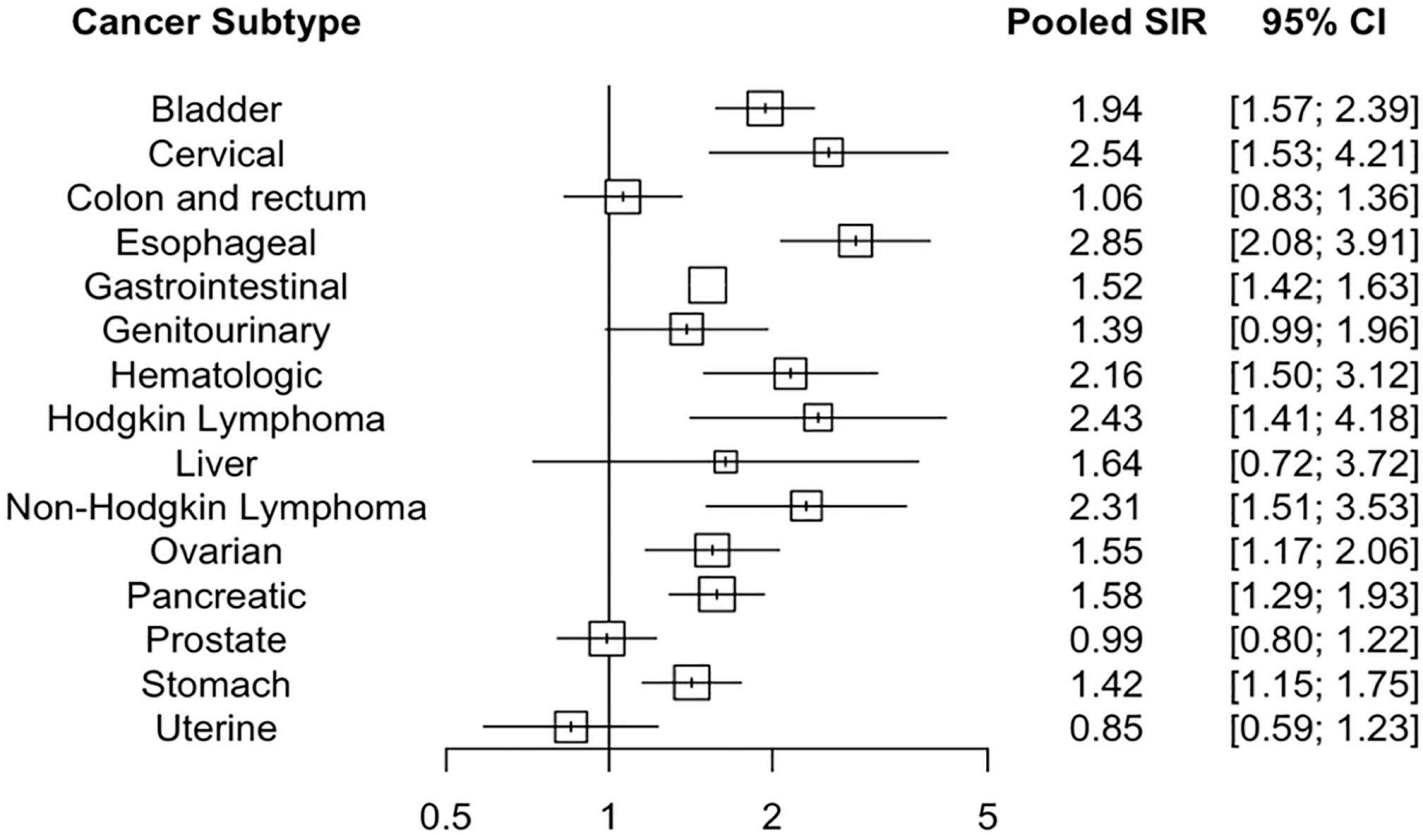
Cancer subtypes. Pooled SIRs for cancer subtypes among people with TB compared to age- and sex- standardized general populations or matched controls.

The pooled SIR for all other cancer subtypes is presented in Fig 4 and the individual forest plots for each cancer subtype are presented in the Appendix (S3A–S3O Fig). TB was associated with an increased pooled SIR of all hematologic cancer (RR 2.16, 95% CI 1.49–3.12, I^2^ = 88%), non-Hodgkin lymphoma (RR 2.31, 95% CI 1.52–3.53, I^2^ = 85%), and Hodgkin lymphoma (RR 2.43, 95% CI 1.41–4.18, I^2^ = 56%). Additionally, TB was associated with an increased pooled SIR of esophageal (RR 2.85, 95% CI 2.09–3.91, I^2^ = 75%), pancreatic (RR 1.58, 95% CI 1.29–1.93, I^2^ = 0%), and stomach (RR 1.42, 95% CI 1.15–1.75, I^2^ = 38%) cancers.

### Risk of cancer over time

**Fig 5:**
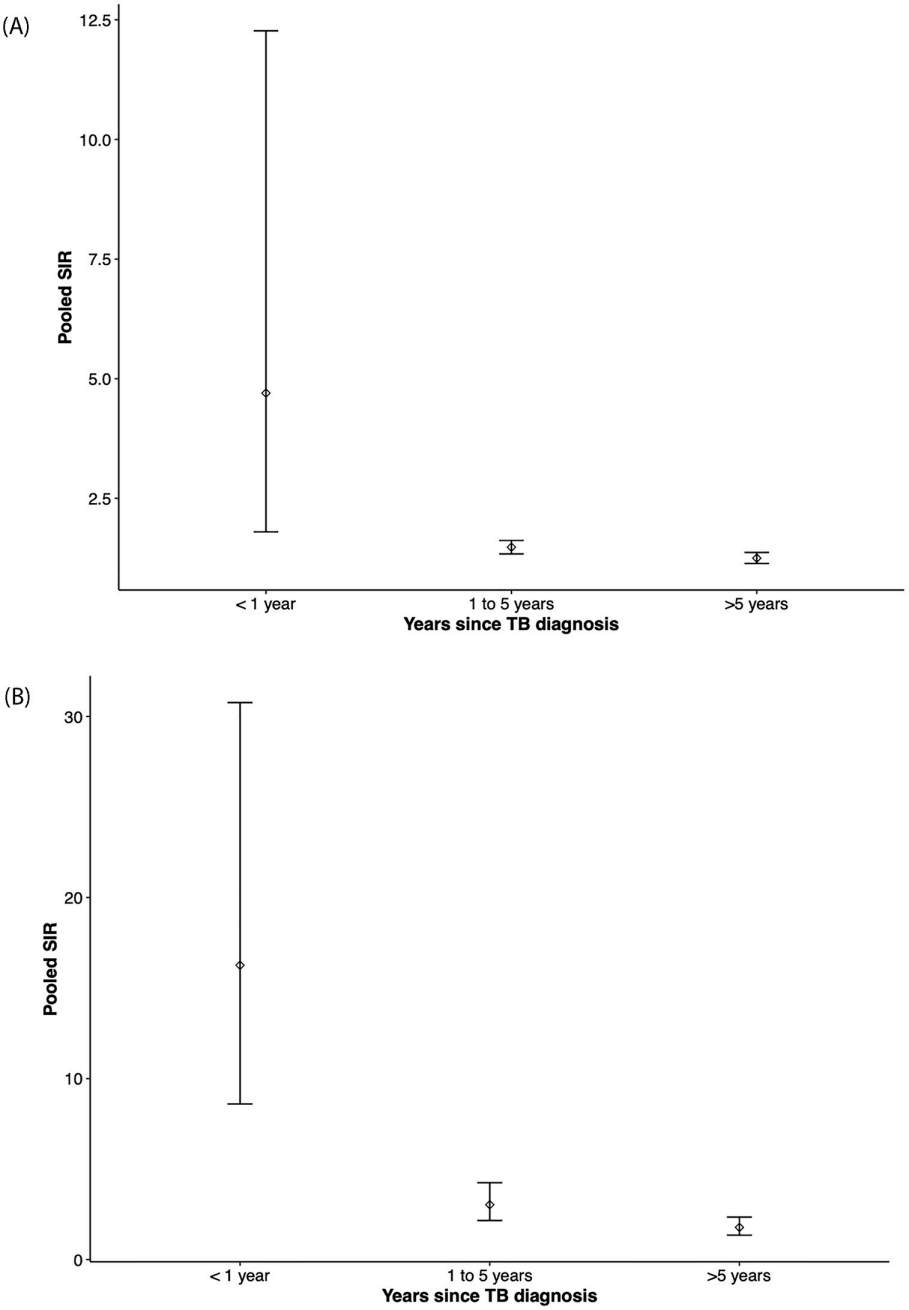
**A. Cancer risk over time.** Pooled SIRs for all cancer subtypes over time among people with TB compared to age- and sex- standardized general populations or matched controls. **B. Lung cancer risk over time.** Pooled SIRs for lung cancer over time among people with TB compared to age- and sex- standardized general populations or matched controls.

Three studies were included in the meta-analysis to determine the risk of cancer over time following TB diagnosis shown in Fig 5A [28, 30, 33]. Of note, the pooled SIR for the less than one year period in overall malignancy and lung cancer included data from Simonsen *et al*. [30] which measured risk of cancer at less than three months after TB diagnosis. The SIR of all cancer was highest within the first year following TB (SIR 4.70, 95% CI 1.80–12.27, I^2^ = 99%). The SIR remained elevated between one to five years (SIR 1.48, 95% CI 1.34–1.62, I^2^ = 62%) and after five years post-TB diagnosis (SIR1.25, 95% CI 1.14–1.37, I^2^ = 59%).

Four studies were included in the meta-analysis to determine the risk of lung cancer over time following TB diagnosis presented in Fig 5B [24, 28, 30, 32]. The pooled SIR of lung cancer followed a similar trend as all cancers. The SIR was highest at less than one year (SIR 16.26, 95% CI 8.60–30.77, I^2^ = 98%), remaining elevated at one to five years post-TB (SIR 3.03, 95% CI 2.16–4.25, I^2^ = 85%) and greater than five years following TB diagnosis (SIR 1.78, 95% CI 1.35–2.35, I^2^ = 54%). When Simonsen *et al*., [30] was removed from the less than one year analysis, the risk of cancer remained elevated but with lower heterogeneity (SIR 11.51, 95% CI 9.27–14.29, I^2^ = 61%).

## Discussion

This systematic review and meta-analysis found that people with TB have an increased risk of both pulmonary and non-pulmonary cancers compared to the general population or suitable controls. Sensitivity and cancer subtype analyses yielded similar conclusions. Relative lung cancer risk following TB remained significantly elevated even when data was stratified by smoking status. We found the risk of all cancers and lung cancer to be highest within the first year following diagnosis of TB. This increased risk of lung cancer persisted for over five years. The level of heterogeneity was high in all pooled risk estimates, despite multiple subset analyses.

To our knowledge, this is the first systematic review to examine the risk of both pulmonary and non-pulmonary cancers at different time periods following TB. Our findings of increased cancer risk among people with TB are consistent with the literature. In 2009, a systematic review by Liang *et al*. found an almost two-fold increase in risk of lung cancer in people with TB [39]. They also found that the risk of lung cancer was over 10 times greater in the first 5 years following TB diagnosis, remaining elevated for more than 20 years after TB [39]. More recent systematic reviews found similar results in pulmonary [11, 40] and non-pulmonary cancers [12] post-TB. In these studies, pooled risk of lung cancer ranged from 1.96 to 2.17 [11, 12, 40]. However, these systematic reviews all included studies with self-report data, which carry a critically high risk of recall bias.

The increased risk of cancer in the TB population may be explained by a temporal bias from reverse causality, with undiagnosed early-stage cancer potentially predisposing people to TB disease. The risk of TB disease may be higher in people with cancer for multiple reasons. Malignancy may induce malnutrition and systemic or local immunocompromise [41, 42]. Cancer treatments including chemotherapy, radiation therapy, and immunotherapy may also predispose to TB disease [43, 44]. Newer cancer therapies such as immune checkpoint inhibitors have been increasingly linked to the diagnosis of TB as well [45]. Specifically, people taking programmed cell death-1 (PD-1) inhibitor drugs have developed severe TB infection thought to be due to enhanced immune system response, not unlike immune-reconstitution inflammatory syndrome in HIV [46].

The higher risk of TB in people with cancer is well-described in the literature. A systematic review and meta-analysis by Dobler *et al*. found that patients with hematological or solid-organ cancers had an approximately two-fold increased risk of TB compared to controls [47]. In lung cancer, the risk of TB was approximately six-fold greater [47]. Studies have demonstrated that the period associated with the greatest risk of TB in patients with cancer is in the first year after cancer diagnosis [48, 49]. Shen *et al*. showed that this high-risk period for TB extends to the year before and after cancer diagnosis [50], which is in line with our findings. To minimize bias due to reverse causality, all studies included in our meta-analysis excluded participants who had previously been diagnosed with cancer.

In addition to reverse causality, the association of TB with cancer risk may be confounded by risk factors for cancer, including smoking, indoor air pollution, HIV, alcohol misuse, and socioeconomic disadvantage [10, 51]. These variables were not controlled for in most of the studies included in our meta-analyses and could impart residual confounding on the risk of cancer in the TB population, potentially persisting for years. Other confounders include surveillance bias: a heightened interaction with healthcare and imaging studies following TB diagnosis. Finally, cancer may be misdiagnosed as TB both clinically and radiographically since both can present with weight loss, cough, hemoptysis, and shortness of breath and appear similar on chest imaging [52]. Reverse causality, increased surveillance, and misdiagnosis may explain some of the high short-term risk of cancer; however, these factors are unlikely to explain the persistently elevated lung cancer risk for over five years following TB. We recommend that future studies include the timing and staging of cancer in their analysis as well as pre- and post-treatment radiographs confirming the resolution of abnormalities attributed to TB.

The pathogenesis underlying the increased malignancy risk following infection with *Mycobacterium tuberculosis* remains unknown, but several hypotheses exist. TB may promote oncogenesis through chronic inflammation, specifically increased circulating levels of tumor necrosis factor-alpha (TNF-α) (9). TNF-α promotes tumor-cell survival via anti-apoptotic intracellular signalling pathways, angiogenesis, and mutagenesis [53]. TB infection leads to fibrous scar formation in the lung, which has been associated with increased cancer over time [54]. This is thought to be due in part to impaired lymphatic flow causing decreased immune surveillance of the area and increased deposition of metastatic cells [55].

There is now evidence to support lung cancer screening in high-risk populations. The Nederlands-Leuvens Longkander Screenings Onderzoek (NELSON) trial showed that lung cancer mortality was 24% lower in high-risk participants who underwent repeated computed tomographic (CT) screening compared with those who did not attend [56]. In the NELSON trial, the cumulative incidence of lung cancer was 5.58 cases per 1,000 person-years in the screening group and 4.91 cases per 1,000 person-years in controls [56]. We found that the cumulative incidence of lung cancer among people with TB ranged from 2.85 to 5.50 cases per 1,000 person-years in studies that did not stratify for smoking (S7 Table). Although limited to only one study, Shiels *et al*. found that the incidence rate for males who smoke diagnosed with TB was much higher, at 17.86 per 1,000 person years [27]. This is in keeping with our pooled data showing a three-fold increased risk of lung cancer in people with TB who smoke compared to people without TB who smoke. These findings suggest that TB and smoking may act synergistically increasing the risk of lung cancer. However, key limitations, namely high heterogeneity, and small sample sizes, lends insufficient evidence to recommend lung cancer screening in TB populations currently. Further studies are required to guide cancer screening in people with TB, particularly those with additional risk factors such as smoking.

The timing of malignancy screening should be informed by the latency period between exposure to risk factors and incident cancer. Current lung cancer screening guidelines focus on smoking as the main risk factor for lung cancer [56–58]. Modelling studies estimate the time gap or lag period between smoking exposure to lung cancer mortality to be 10 to 30 years [59, 60]. The latency periods for occupational cancer risk factors such as asbestos, silica, and diesel exhaust are also estimated to be on the order of decades [61–63]. Less is known about the temporal relationship between particulate pollution and lung cancer [64]. While our findings indicate that the risk of lung cancer remains elevated for over five years following diagnosis with TB, further research is needed to determine the latency period between TB disease and cancer to inform screening guidelines in this population. Based on our findings, and in the absence of further evidence to support more aggressive screening, we suggest people with TB undergo age-appropriate cancer screenings. Clinicians should maintain a high degree of suspicion for pulmonary and non-pulmonary cancers, especially in the first year following TB diagnosis and in people who smoke. We recommend considering post-treatment imaging to ensure improvement or resolution of radiographic abnormalities attributed to TB. Any persistent or worsening abnormalities should be investigated for malignancy.

There are strengths and limitations to this systematic review. Our main strength was that we provided pooled risk estimates for both pulmonary and non-pulmonary cancers from diverse populations with various effect estimates and cancer risk over time. Compared to other systematic reviews on TB and cancer [11, 12, 39, 40], we used strict inclusion criteria for the diagnosis of both TB and cancer, requiring either clinical or health administrative diagnoses. For our pooled risk analyses calculated with SIRs, we chose studies with more robust diagnostic criteria for TB, with two studies reporting 47% [32, 33] and 58% [30] culture-confirmed TB, and one study requiring medical database codes plus prescription of anti-TB medication [28]. These three studies together represented approximately 60% of the effect estimates for the pooled risk analyses for all cancers and lung cancer. Finally, all included studies accounted for at least age, sex, or geographic location, with most controlling for additional variables.

The limitations of our study are predominantly related to the use of published data. The included studies used a wide range of control or reference populations to calculate risk estimates. Because we do not have access to the raw data, we were unable to account for all clinical and population factors that influence cancer risk. Most importantly, many studies, including all five studies from Taiwan, did not control for smoking status. Despite this, we were able to meta-analyze data stratified by smoking status. Other sources of potential bias were lack of control for other comorbid conditions such as COPD and HIV. Although we collected data that stratified or controlled for these and other comorbid conditions, the low sample size precluded pooled or sensitivity analyses. Also, outcome measures in our review did not uniformly control for bias, contributing to the variability in effect estimates. For example, studies with SIRs only accounted for age, sex, and geographic location. However, pooled risk calculated with HRs, which controlled for more variables, yielded similar results. Moreover, heterogeneity overall remained high thus our findings need to be interpreted with caution. Other limitations are the relatively small sample sizes for pooled analyses and the inclusion of only English language studies. Although our review included studies from nine countries, the study populations were predominantly high-income, with only two studies originating from the thirty countries with the highest TB burden according to the WHO [2]. Taken together, these limitations and the observational nature of the included studies prevent causal inferences about cancer risk following TB.

In conclusion, our results demonstrate that people diagnosed with TB have an increased risk of both pulmonary and non-pulmonary cancers. The risk is highest in the first year following diagnosis with TB but appears to persist for years after TB treatment is complete. Further research is required to determine if the increased risk of cancer in TB survivors is causal or correlational. In addition, there is insufficient evidence to recommend broad or site-specific malignancy screening among people with TB; however, clinicians should have a high index of suspicion for cancer in this population, particularly for pulmonary malignancies in the first year following TB diagnosis.

## Supporting information

S1 Table MEDLINE, EMBASE, and Cochrane database search for systematic review of tuberculosis and risk of cancer. (DOCX)

S2 Table Risk of bias criteria. Predefined criteria for each domain of bias using Risk of Bias in Non-randomized Studies of Interventions (ROBINS-I) tool. HR = hazard ratio, ICD = International Classification of Diseases, IR = incidence rate, OR = odds ratio, TB = tuberculosis disease.
(DOCX)

S3 Table Adjusted variables. Description of adjustment variables for hazard ratios in systematic review. *Studies included in meta-analysis.
(DOCX)

S4 Table Population characteristics. Population characteristics of included studies.
(DOCX)

S5 Table Characteristics of TB diagnosis. Characteristics of TB diagnosis and treatment of included studies.
(DOCX)

S6 Table Characteristics of cancer diagnosis. Characteristics of cancer diagnosis and treatment of included studies.
(DOCX)

S7 Table Cumulative incidence of lung cancer. *Data stratified for male smokers with TB.
(DOCX)

S1 Fig All cancer stratified males and females. Meta-analysis of the SIR for overall cancer among males with TB compared to age- and sex- standardized general populations or matched controls.
(TIF)

S2 Fig Lung cancer smoking stratified. Meta-analysis of the SIR for lung cancer among people with TB compared to age- and sex- standardized general populations or matched controls, stratified for smoking.
(TIF)

S3 Fig Cancer by subtype. Meta-analysis of the SIR for cancer subtypes among people with TB compared to age- and sex- standardized general populations or matched controls.
(TIF)

S4 Fig Cancer risk over time < 1 year, 1–5 years, > 5 years. Pooled SIRs of all cancer subtypes during first year from diagnosis, 1–5 years from diagnosis and > 5 years from diagnosis among people with TB compared to age- and sex- standardized general populations or matched controls.
(TIF)

S5 Fig Lung cancer over time < 1 year, 1–5 years, > 5 years. Pooled SIRs of lung cancer during first year from diagnosis, 1–5 years from diagnosis and > 5 years from diagnosis among people with TB compared to age- and sex- standardized general populations or matched controls.
(TIF)

S1 Checklist PRISMA 2020 checklist. (DOCX)
