# Supplementary material for: Tuberculosis and risk of cancer: A systematic review and meta-analysis
Source: PLoS One. 2022 Dec 30;17(12):e0278661. doi: 10.1371/journal.pone.0278661 (PMC9803143; doi:10.1371/journal.pone.0278661)
Supplement: S1 Table — (DOCX) [file pone.0278661.s001.docx]

**S1 Table. MEDLINE, EMBASE and Cochrane search strategy**

| **Number** | **Search** | **Records** |
| --- | --- | --- |
| 1 | exp *Tuberculosis/ | 176044 |
| 2 | Tuberculosis.ti,ab. | 198863 |
| 3 | (TB or "mycobacterium tuberculosis" or "mycobacterial infection*").ti,ab. | 100284 |
| 4 | Or/1-3 | 265740 |
| 5 | exp Neoplasms/ | 3534332 |
| 6 | cancer.mp. | 1912086 |
| 7 | malignan*.mp. | 624326 |
| 8 | neoplas*.mp. | 3084703 |
| 9 | Or/5-8 | 4258690 |
| 10 | exp randomized controlled trial/ | 544738 |
| 11 | exp case-control studies/ | 1222211 |
| 12 | exp clinical study/ | 1026894 |
| 13 | exp Cohort Studies/ | 2207343 |
| 14 | (random* or cohort or case control or RCT or population-based stud* or incidence stud* or follow-up stud*).mp. | 3014232 |
| 15 | or/10-14 | 4268435 |
| 16 | 4 and 9 | 18366 |
| 17 | 15 and 16 | 3249 |
| 18 | (exp animal/ or nonhuman/) not exp human/ | 4885466 |
| 19 | 17 not 18 | 3214 |

**S1A Table:** MEDLINE database search for systematic review of tuberculosis and risk of cancer.

| **Number** | **Search** | **Records** |
| --- | --- | --- |
| 1 | exp tuberculosis/ | 201741 |
| 2 | tuberculosis.ti,ab. | 191251 |
| 3 | (TB or "mycobacterium tuberculosis" or "mycobacterial infection*").ti,ab. | 119152 |
| 4 | or/1-3 | 273154 |
| 5 | exp neoplasm/ | 4803664 |
| 6 | cancer.mp. | 3896963 |
| 7 | malignan*.mp. | 982664 |
| 8 | neoplas*.mp. | 1023967 |
| 9 | or/5-8 | 5673640 |
| 10 | exp randomized controlled trial/ | 676805 |
| 11 | exp case control study/ | 1945726 |
| 12 | exp cohort analysis/ | 751458 |
| 13 | exp population research/ | 117091 |
| 14 | or/10-13 | 1670183 |
| 15 | 4 and 9 | 45900 |
| 16 | 14 and 15 | 2273 |

**S1B Table:** EMBASE database search for systematic review of tuberculosis and risk of cancer.

| **Number** | **Search** | **Records** |
| --- | --- | --- |
| 1 | exp Tuberculosis/ | 2478 |
| 2 | tuberculosis.ti,ab. | 6405 |
| 3 | (TB or "mycobacterium tuberculosis" or "mycobacterial infection*").ti,ab. | 4164 |
| 4 | or/1-3 | 8166 |
| 5 | exp Neoplasms/ | 83665 |
| 6 | cancer.mp. | 185431 |
| 7 | malignan*.mp. | 30904 |
| 8 | neoplas*.mp. | 89193 |
| 9 | or/5-8 | 229328 |
| 10 | exp Randomized Controlled Trial/ | 131 |
| 11 | randomized control* trial.mp. | 586693 |
| 12 | exp Case-Control Studies/ | 14716 |
| 13 | clinical study.mp. | 325478 |
| 14 | exp Cohort Studies/ | 156694 |
| 15 | (random* or cohort or case control or RCT or population-based stud* or incidence stud* or follow-up stud*).mp. | 1254474 |
| 16 | or/10-15 | 1309242 |
| 17 | 4 and 9 | 805 |
| 18 | 16 and 17 | 685 |

**S1C Table:** Cochrane Central Register of Controlled Trials database search for systematic review of tuberculosis and risk of cancer.
