# Supplementary material for: Tuberculosis and risk of cancer: A systematic review and meta-analysis
Source: PLoS One. 2022 Dec 30;17(12):e0278661. doi: 10.1371/journal.pone.0278661 (PMC9803143; doi:10.1371/journal.pone.0278661)
Supplement: S2 Table — Predefined criteria for each domain of bias using Risk of Bias in Non-randomized Studies of Interventions (ROBINS-I) tool. HR = hazard ratio, ICD = International Classification of Diseases, IR = incidence rate, OR = odds ratio, TB = tuberculosis disease. (DOCX) [file pone.0278661.s002.docx]

| **Domain of bias** | **Low** | **Moderate** | **Serious** | **Critical** |
| --- | --- | --- | --- | --- |
| **Confounding** | Randomized or quasi-experimental study design | Controlled for at least one demographic variable (e.g. age or sex) and smoking | Controlled for a demographic variable (e.g. age or sex) or smoking | Not controlled for any demographic variable or medical risk factor |
| **Selection and intervention classification** | TB confirmed in validated database with greater than 50% of cases microbiologically diagnosed | TB confirmed clinically with ICD codes, medical database, or physician diagnosis | TB confirmed by radiograph only | TB confirmed by self-report or other low-quality diagnostic criteria |
| **Missing data** | Less than 10% of data missing or lost to follow up | Missing data and loss to follow up not reported, study used a validated and nationally representative database | Missing data and loss to follow up not reported, study did not use a validated or nationally representative database | Greater than 30% of data is missing or lost to follow up |
| **Measurement of outcome** | Cancer confirmed by histology or cytology | Cancer confirmed clinically with ICD codes, medical database, or physician diagnosis | Cancer confirmed clinically with ICD codes, medical database, or physician diagnosis; subjects with previous cancer not excluded | Cancer confirmed by self-report or other low-quality diagnostic criteria; subjects with previous cancer not excluded |
| **Selection of reported result** | IR or adjusted HR | SIR or OR | No adjusted outcome measure | Outcome measure not applicable to research question |
| **Overall** | Lowest score in any subgroup | | | |

**Supplemental Table 2.** Predefined criteria for each domain of bias using the Risk of Bias in Non-randomized Studies of Interventions (ROBINS-I) tool.

HR = hazard ratio, ICD = International Classification of Diseases, IR = incidence rate, OR = odds ratio, TB = tuberculosis disease
