# Supplementary material for: Tuberculosis and risk of cancer: A systematic review and meta-analysis
Source: PLoS One. 2022 Dec 30;17(12):e0278661. doi: 10.1371/journal.pone.0278661 (PMC9803143; doi:10.1371/journal.pone.0278661)
Supplement: S3 Table — Description of adjustment variables for hazard ratios in systematic review. *Studies included in meta-analysis. (DOCX) [file pone.0278661.s003.docx]

**S2 Table. Description of adjustment variables for hazard ratios in systematic review.**

| **Author** | **Adjustment variables** |
| --- | --- |
| Yu *et al.* (2011) | Cox proportional hazards model with multivariate model controlled for comorbidities including hypertension, dyslipidemia, diabetes, and COPD |
| Wu *et al.* (2011) | Cox proportional hazards model with variables age, sex, TB infection, diabetes, chronic renal failure, autoimmune diseases, and COPD |
| Shiels *et al.* (2011)* | Proportional hazards regression model adjusted for age and baseline cigarette use |
| Huang *et al.* (2015)* | Cox proportional hazards model controlled for age, geographical area, urbanization level, low income, and comorbidities |
| Hong *et al.* (2016) | Cox proportional hazards model adjusted for age at enrollment and cigarette smoking status |
| Oh *et al.* (2020) | Cox proportional hazards model adjusted for age, sex, education, income level, smoking status, BMI, and moderate or vigorous physical activity |
| An *et al.* (2020)* | Cox proportional hazards model adjusted for sex, age, household income, smoking status, and TB |
| Park *et al.* (2021) | Cox proportional hazards model adjusted for sex, BMI, smoking status, CCI score, and income percentile |

*Study included in meta-analysis.
