# Supplementary material for: Tuberculosis and risk of cancer: A systematic review and meta-analysis
Source: PLoS One. 2022 Dec 30;17(12):e0278661. doi: 10.1371/journal.pone.0278661 (PMC9803143; doi:10.1371/journal.pone.0278661)
Supplement: S4 Table — Population characteristics of included studies. (DOCX) [file pone.0278661.s004.docx]

**S3 Table. Population characteristics of included studies.**

| **Author** | **Country income** | **Average age (years)** | **% Female** | **% Smokers** | **% COPD** |
| --- | --- | --- | --- | --- | --- |
| Doody *et al.* (1992) | High income | 47 at study entry and 63 at cancer diagnosis (mean) | Under 40% | Not reported | Not reported |
| Askling *et al.* (2001) | High income | Not reported | 47% | Not reported | Not reported |
| Yu *et al.* (2011) | High Income | Not reported | 42% | Not reported | 44% |
| Wu *et al.* (2011) | High income | 69 for TB and 72 for controls at study entry (median) | 32% | Not reported | 49% |
| Shiels *et al.* (2011) | High income | 57 at study entry (median) | 0% | 100% | 55% |
| Kuo *et al.* (2013) | High income | 64 at TB diagnosis (median) | 69% | Not reported | Not reported |
| Lien *et al.* (2013) | High income | 58 at study entry (mean) | Not reported | Not reported | Not reported |
| Simonsen *et al.* (2014) | High income | 43 at TB diagnosis (median) | 32% | Not reported | 93% |
| Kristinsson *et al.* (2015) | High income | 47 at cancer diagnosis (median) | 41% | Not reported | Not reported |
| Huang *et al.* (2015) | High income | Not reported | 30% of cancer group and 47% of controls | Not reported | 24% of cancer group and 8% of controls |
| Everatt *et al.* (2016) | High income | 47 at TB diagnosis (mean) | 30% | 64% | Not reported |
| Hong *et al.* (2016) | High income | 50 for TB group and 43 for controls at study entry (mean) | 35% | 50% of males with TB and 55% of male controls. Less than 2% of all females. | Not reported |
| Everatt *et al.* (2017) | High income | 47 at TB diagnosis (mean) | 30% | 64% | Not reported |
| Oh *et al.* (2020) | High income | 63 for TB group and 58 for controls at study entry (mean) | 40% of TB group and 53% of controls | 24% of TB group and 22% of controls | Not reported |
| An *et al.* (2020) | High income | Not reported | 41% | 31% of TB group and 29% of controls | Not reported |
| Park *et al.* (2021) | High Income | 66 at study entry (mean) | 31% of TB group and 52% of controls. | 24% of TB group and 19% of controls | 30% of TB group and 27% of controls |
| Chen *et al.* (2021) | Upper middle income | 55 for TB and 41 for controls at study entry (median) | 62% of TB group and 94% of controls. | Not reported | Not reported |
