# Supplementary material for: Tuberculosis and risk of cancer: A systematic review and meta-analysis
Source: PLoS One. 2022 Dec 30;17(12):e0278661. doi: 10.1371/journal.pone.0278661 (PMC9803143; doi:10.1371/journal.pone.0278661)
Supplement: S5 Table — Characteristics of TB diagnosis and treatment of included studies. (DOCX) [file pone.0278661.s005.docx]

**S4 Table. Characteristics of TB diagnosis and treatment of included studies.**

| **Author** | **Source of TB diagnosis** | **TB diagnosis criteria** | **TB location** | **TB treatment** | **TB treatment details** |
| --- | --- | --- | --- | --- | --- |
| Doody *et al.* (1992) | Kaiser Permanente records for California and Oregon, USA (1956-1982) | Medical records and radiographic reports | Not reported | Not reported |  |
| Askling *et al.* (2001) | Two tuberculosis dispensaries and one sanitorium in Sweden (1939-1960) | Medical records | Both | Yes | Medical and surgical treatment. Medications not reported |
| Yu *et al.* (2011) | NHI program research database of Taiwan (1998-2001) | ICD and A-codes from outpatient and inpatient medical records | Pulmonary TB | Not reported |  |
| Wu *et al.* (2011) | NHI program research database of Taiwan (1997-2008) | ICD and A-codes from outpatient and inpatient medical records plus prescription of anti-TB medications | Pulmonary TB | Yes | Prescription of anti-TB medications for at least 28 days |
| Shiels *et al.* (2011) | National Hospital Discharge Register of Finland (1976-1995) | ICD codes from inpatient medical records | Pulmonary TB | Not reported |  |
| Kuo *et al.* (2013) | NHI program research database of Taiwan (2000-2010) | ICD and A-codes from outpatient and inpatient medical records plus prescription of anti-TB medications | Both | Yes | Prescription of anti-TB medications for 2 months |
| Lien *et al.* (2013) | NHI program research database of Taiwan (1998-2008) | ICD and A-codes from outpatient and inpatient medical records plus prescription of anti-TB medications | Both | Yes | Prescriptions consisting of at least 2 anti-TB drugs for over 120 days of a 180 day period, and at least one prescription containing 3 or more anti-TB drugs |
| Simonsen *et al.* (2014) | Danish National Registry of Patients (1978-2011) | ICD codes from first-time hospital contact of active TB, 58% culture confirmed | Both | Not reported |  |
| Kristinsson *et al.* (2015) | Swedish Patient Registry (1964-2004) | Medical records | Pulmonary TB | Not reported |  |
| Huang *et al.* (2015) | NHI program research database of Taiwan (2001-2003) | ICD codes plus more than 2 outpatient visits or one admission | Pulmonary TB | Not reported |  |
| Everatt *et al.* (2016) | Lithuanian TB registry (1998-2012) | Medical records, 47% culture confirmed | Both | Not reported |  |
| Hong *et al.* (2016) | NHIS of Korea (1997-2013) | ICD-10 codes and chest radiographs | Pulmonary TB | Not reported |  |
| Everatt *et al.* (2017) | Lithuanian TB registry (1998-2012) | Medical records, 47% culture confirmed | Both | Not reported |  |
| Oh *et al.* (2020) | KNHANES database (2008-2013) | Medical records and chest radiographs | Pulmonary TB | Not reported |  |
| An *et al.* (2020) | Korean National Health Insurance Service-National Sample Cohort (2003-2013) | ICD codes and prescription of anti-TB medications | Pulmonary TB | Yes | Two or more anti-TB medications prescribed for over 28 days within 90 days of first diagnosis of TB |
| Park *et al.* (2021) | Korean National Health Insurance-Service-National Sample Cohort 2.0 (2002-2015) | Chest radiographs | Pulmonary TB | Not reported |  |
| Chen *et al.* (2021) | Department of TB Registry in Xinjiang Cancer Hospital in China (period not reported) | Medical records | Both | Not reported |  |

ICD = International Classification of Diseases, NHI = National Health Insurance, NHIS = National Health Insurance Service.
