# Supplementary material for: Tuberculosis and risk of cancer: A systematic review and meta-analysis
Source: PLoS One. 2022 Dec 30;17(12):e0278661. doi: 10.1371/journal.pone.0278661 (PMC9803143; doi:10.1371/journal.pone.0278661)
Supplement: S6 Table — Characteristics of cancer diagnosis and treatment of included studies. (DOCX) [file pone.0278661.s006.docx]

**S5 Table. Characteristics of cancer diagnosis and treatment of included studies.**

| **Author** | **Source of cancer diagnosis** | **Cancer diagnosis criteria** | **All cancer** | **Lung cancer** | **Cancer(s) studied** | **Stratified variables** | **Data over time** |
| --- | --- | --- | --- | --- | --- | --- | --- |
| Doody *et al.* (1992) | Kaiser Permanente records for California and Oregon, USA (1956-1982) | Medical records | No | No | Leukemia with subtypes, multiple myeloma, and NHL |  |  |
| Askling *et al.* (2001) | Swedish Cancer Register (1958-1996) | Medical records | Yes | No | All cancers, NHL, and HL |  |  |
| Yu *et al.* (2011) | NHI program research database of Taiwan (1998-2007). | ICD-9-CM codes and A-codes from inpatient and outpatient records | No | Yes | Lung cancer |  |  |
| Wu *et al.* (2011) | Registry for Catastrophic Illness Patient Database of Taiwan (1997-2008) | ICD-9-CM codes and A-codes from inpatient and outpatient records with histologic confirmation required | No | Yes | Lung cancer | Stratified by age, sex, diabetes, chronic kidney disease, and COPD | Lung cancer |
| Shiels *et al.* (2011) | Finnish Cancer Registry (1985-2005) | Medical records | No | Yes | Lung cancer with subtypes |  | Lung cancer |
| Kuo *et al.* (2013) | Registry for Catastrophic Illness Patient Database of Taiwan (2000-2010) | ICD-9-CM codes from inpatient and outpatient records with histologic confirmation required | Yes | Yes | All cancers, head and neck, GI, lung, MSK, skin, breast, GU, CNS, and hematologic | Stratified by sex | All cancer and lung cancer |
| Lien *et al.* (2013) | Registry for Catastrophic Illness Patient Database of Taiwan (1998-2010) | ICD-9-CM codes and A-codes from inpatient and outpatient records with histologic confirmation required | No | No | Urinary tract cancer with subtypes | Stratified by age and sex |  |
| Simonsen *et al.* (2014) | Danish Cancer Registry (1978-2011) | Medical records | Yes | Yes | All cancers, head and neck, GI, lung, MSK, skin, breast, GU, CNS, and hematologic | Stratified by age, sex, TB location, COPD, alcoholism, CCI score, HIV, and cancer etiology | All cancer and lung cancer |
| Kristinsson *et al.* (2015) | Swedish Cancer Register (1965-2004) | Medical records | No | No | HL |  |  |
| Huang *et al.* (2015) | Taiwan Cancer Registry Database (2004-2008) | ICD-10 codes and A-codes for patient encourters with histologic confirmation by ICD-O codes | No | Yes | Lung cancer with subtypes | Stratified by sex |  |
| Everatt *et al.* (2016) | Lithuanian Cancer Registry (1998-2012) | ICD-10 codes from the national cancer registry | No | Yes | Lung cancer with subtypes | Stratified by smoking and TB location | Lung cancer |
| Hong *et al.* (2016) | NHIS of Korea (1997-2013) | ICD-10 codes from national health data registry and at least two or more hospitalizations for lung cancer | No | Yes | Lung cancer |  |  |
| Everatt *et al.* (2017) | Lithuanian Cancer Registry (1998-2012) | ICD-10 codes from the national cancer registry | Yes | No | All cancers, head and neck, GI, MSK, breast, skin, CNS, hematologic | Stratified by sex | All cancer |
| Oh *et al.* (2020) | Korea Central Cancer Registry (2008-2013) | ICD-10 codes from the national cancer registry | No | Yes | Lung cancer with subtypes | Stratified by age, sex, and smoking status |  |
| An *et al.* (2020) | Korean National Health Insurance Service-National Sample Cohort (2003-2013) | ICD-10 codes from national health data registry | No | Yes | Lung cancer | Stratified by age | Lung cancer |
| Park *et al.* (2021) | Korean National Health Insurance-Service-National Sample Cohort 2.0 (2002-2015) | ICD-10 codes from a national health data registry present more than three times in one year or an inpatient hospitalization | No | Yes | Lung cancer | Stratified by age and COPD |  |
| Chen *et al.* (2021) | Xinjiang Cancer Hospital (2016-2018) | Medical records | Yes | Yes | All cancers, head and neck, lung, GI, MSK, breast, skin, CNS, and hematologic | Stratified by sex |  |

CNS = central nervous system, CCI = Charlson Comorbidity Index, COPD = chronic obstructive pulmonary disease, GI = gastrointestinal, GU = genitourinary, HIV = human immunodeficiency virus, ICD = International Classification of Diseases, MSK = musculoskeletal, NHI = National Health Insurance, NHIS = National Health Insurance Service.
