# Supplementary material for: Tuberculosis and risk of cancer: A systematic review and meta-analysis
Source: PLoS One. 2022 Dec 30;17(12):e0278661. doi: 10.1371/journal.pone.0278661 (PMC9803143; doi:10.1371/journal.pone.0278661)
Supplement: S7 Table — *Data stratified for male smokers with TB. (DOCX) [file pone.0278661.s007.docx]

**S6 Table. Cumulative incidence of lung cancer.**

| **Author** | **Number of patients with cancer and TB** | **Person years** | **Incidence per 1,000 person years** |
| --- | --- | --- | --- |
| Shiels et al. (2011)* | 44 | 2,463 | 17.86 |
| Kuo et al. (2013) | 159 | 28,866 | 5.51 |
| Simonsen et al. (2014) | 429 | 150,400 | 2.85 |
| Everatt et al. (2016) | 477 | 138,811 | 3.44 |
| An et al. (2020) | 86 | 20,423 | 4.21 |

*Data stratified for male smokers with TB.
