# Supplementary figures and images for: Tuberculosis and risk of cancer: A systematic review and meta-analysis
Source: PLoS One. 2022 Dec 30;17(12):e0278661. doi: 10.1371/journal.pone.0278661 (PMC9803143; doi:10.1371/journal.pone.0278661)

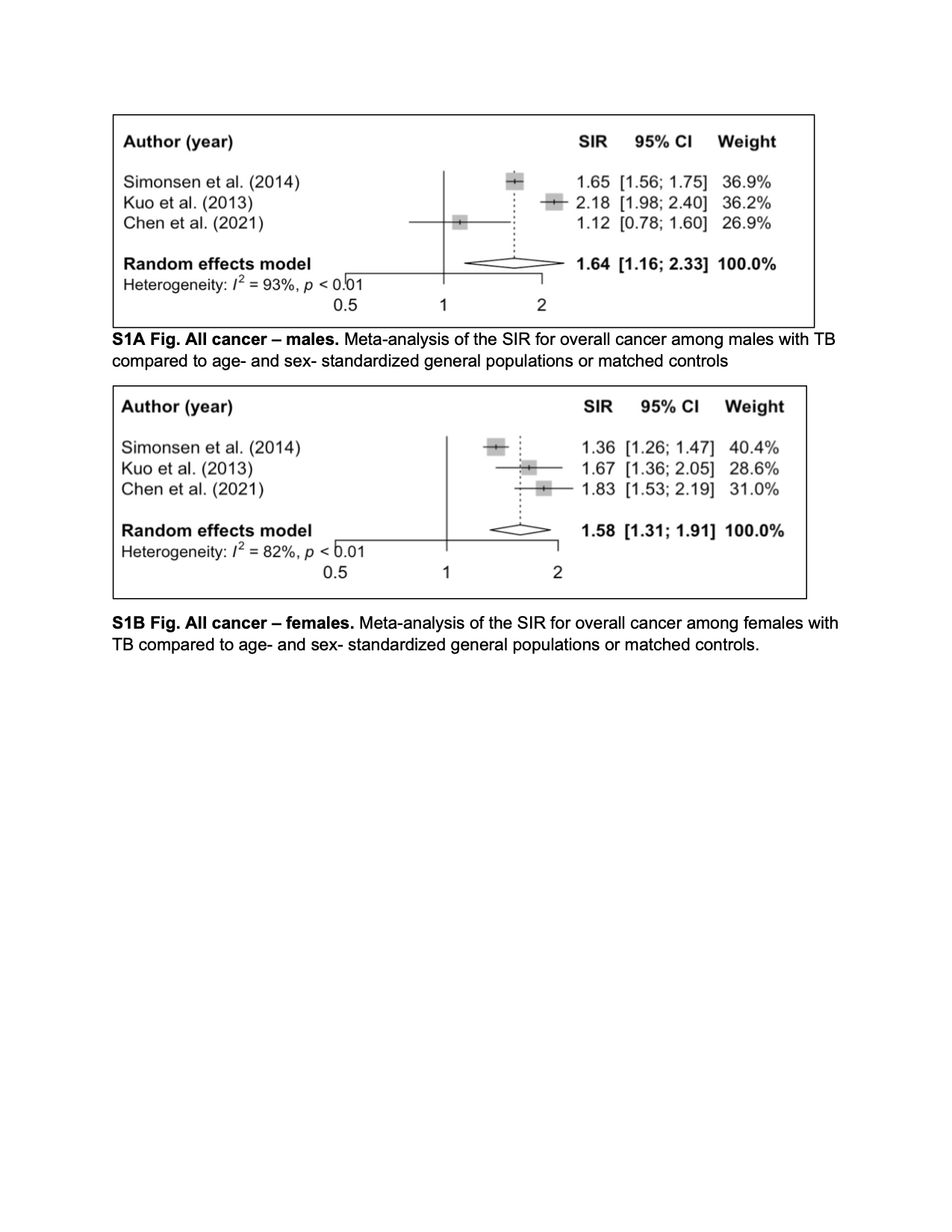

Supplement: S1 Fig — Meta-analysis of the SIR for overall cancer among males with TB compared to age- and sex- standardized general populations or matched controls. (TIF) [file pone.0278661.s008.tif]

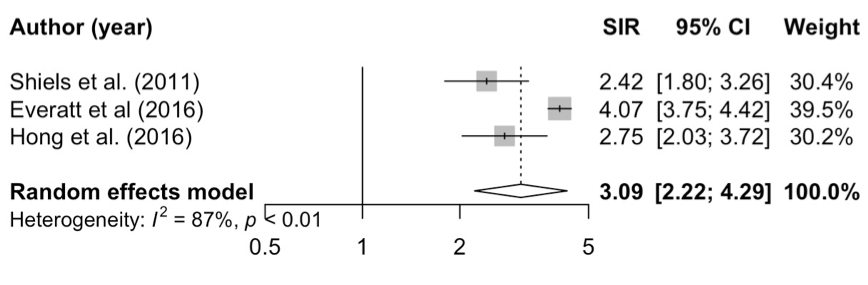

Supplement: S2 Fig — Meta-analysis of the SIR for lung cancer among people with TB compared to age- and sex- standardized general populations or matched controls, stratified for smoking. (TIF) [file pone.0278661.s009.tif]

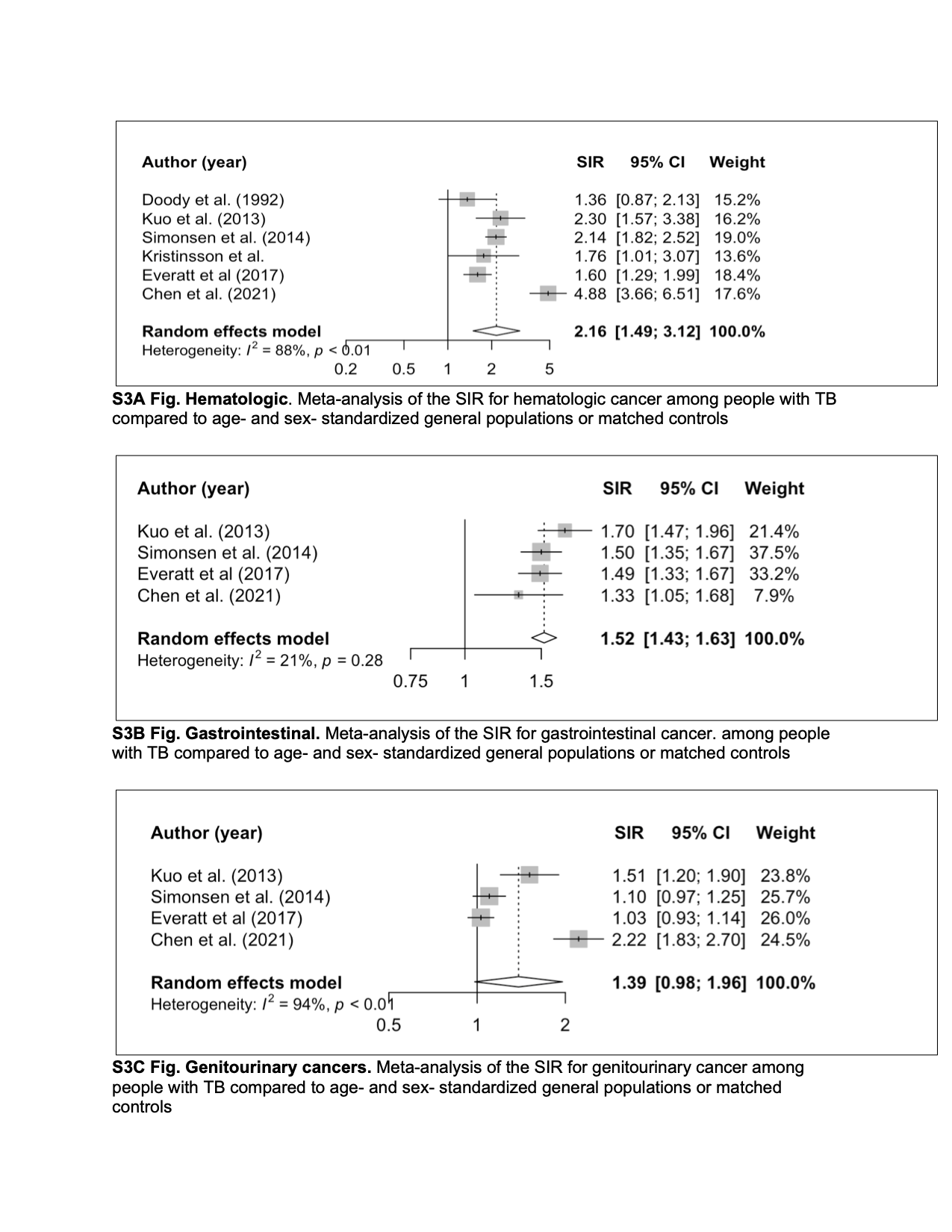

Supplement: S3 Fig — Meta-analysis of the SIR for cancer subtypes among people with TB compared to age- and sex- standardized general populations or matched controls. (TIF) [file pone.0278661.s010.tif]

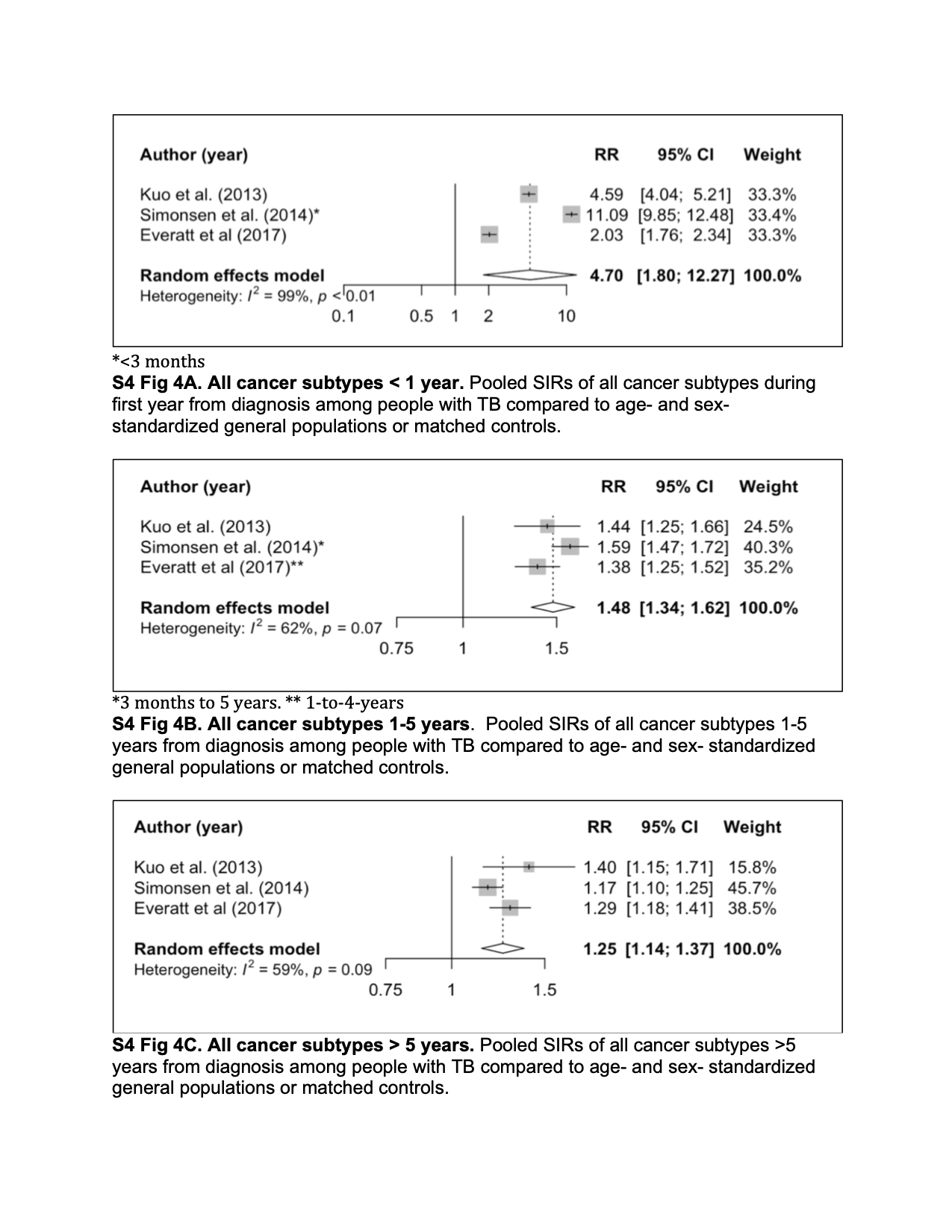

Supplement: S4 Fig — Pooled SIRs of all cancer subtypes during first year from diagnosis, 1–5 years from diagnosis and > 5 years from diagnosis among people with TB compared to age- and sex- standardized general populations or matched controls. (TIF) [file pone.0278661.s011.tif]

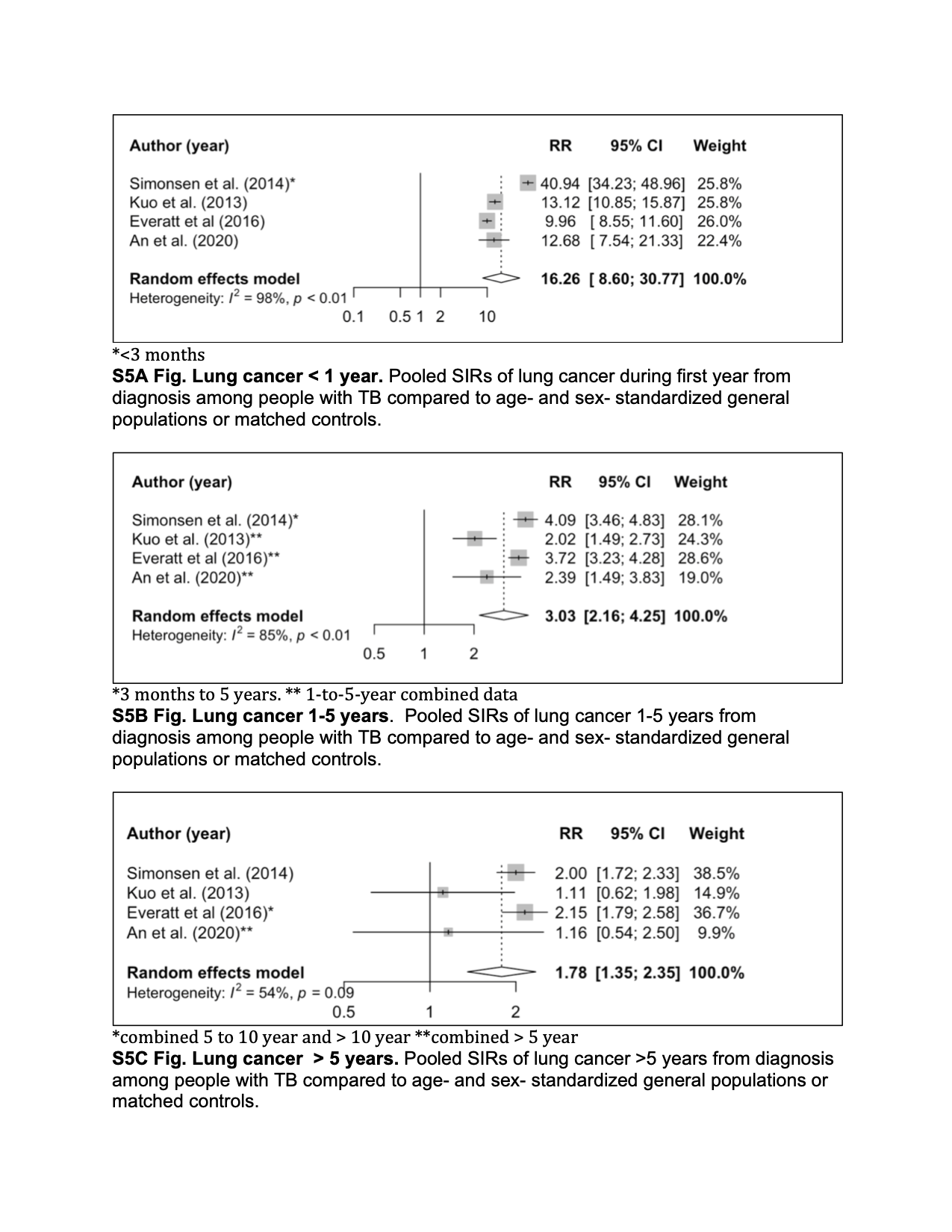

Supplement: S5 Fig — Pooled SIRs of lung cancer during first year from diagnosis, 1–5 years from diagnosis and > 5 years from diagnosis among people with TB compared to age- and sex- standardized general populations or matched controls. (TIF) [file pone.0278661.s012.tif]
